# Natural product rhynchophylline prevents stress-induced hair graying by preserving melanocyte stem cells via the β2 adrenergic pathway suppression

**DOI:** 10.1007/s13659-023-00421-z

**Published:** 2023-12-01

**Authors:** Xinxin Li, Runlu Shi, Lingchen Yan, Weiwei Chu, Ruishuang Sun, Binkai Zheng, Shuai Wang, Hui Tan, Xusheng Wang, Ying Gao

**Affiliations:** 1 School of Pharmaceutical Sciences (Shenzhen), Sun Yat-sen University, Shenzhen, 518107 China; 2 Department of Anesthesiology, The First Affiliated Hospital of Bengbu Medical College, Bengbu, 233004 China; 3 Center for Child Care and Mental Health, Shenzhen Children’s Hospital Affiliated to Shantou University Medical College, Shenzhen, 518026 China; 4 Institute of Biopharmaceutical and Health Engineering, Tsinghua Shenzhen International Graduate School, Tsinghua University, Shenzhen, 518055 China; 5 Department of Plastic and Reconstructive Surgery, Guangdong Second Provincial General Hospital, Guangzhou, 510317 China; 6 The Yonghe Medical Beauty Clinic Department, Guangzhou, 510630 China; 7 Department of Anesthesiology, The First People’s Hospital of Foshan, Foshan, 528000 China

**Keywords:** Natural product, Rhynchophylline, Stress, Hair graying, β2 adrenergic signaling

## Abstract

Norepinephrine (NA), a stress hormone, can accelerate hair graying by binding to β2 adrenergic receptors (β_2_AR) on melanocyte stem cells (McSCs). From this, NA-β_2_AR axis could be a potential target for preventing the stress effect. However, identifying selective blockers for β_2_AR has been a key challenge. Therefore, in this study, advanced computer-aided drug design (CADD) techniques were harnessed to screen natural molecules, leading to the discovery of rhynchophylline as a promising compound. Rhynchophylline exhibited strong and stable binding within the active site of β_2_AR, as verified by molecular docking and dynamic simulation assays. When administered to cells, rhynchophylline effectively inhibited NA-β_2_AR signaling. This intervention resulted in a significant reduction of hair graying in a stress-induced mouse model, from 28.5% to 8.2%. To gain a deeper understanding of the underlying mechanisms, transcriptome sequencing was employed, which revealed that NA might disrupt melanogenesis by affecting intracellular calcium balance and promoting cell apoptosis. Importantly, rhynchophylline acted as a potent inhibitor of these downstream pathways. In conclusion, the study demonstrated that rhynchophylline has the potential to mitigate the negative impact of NA on melanogenesis by targeting β_2_AR, thus offering a promising solution for preventing stress-induced hair graying.

**Graphical Abstract:**

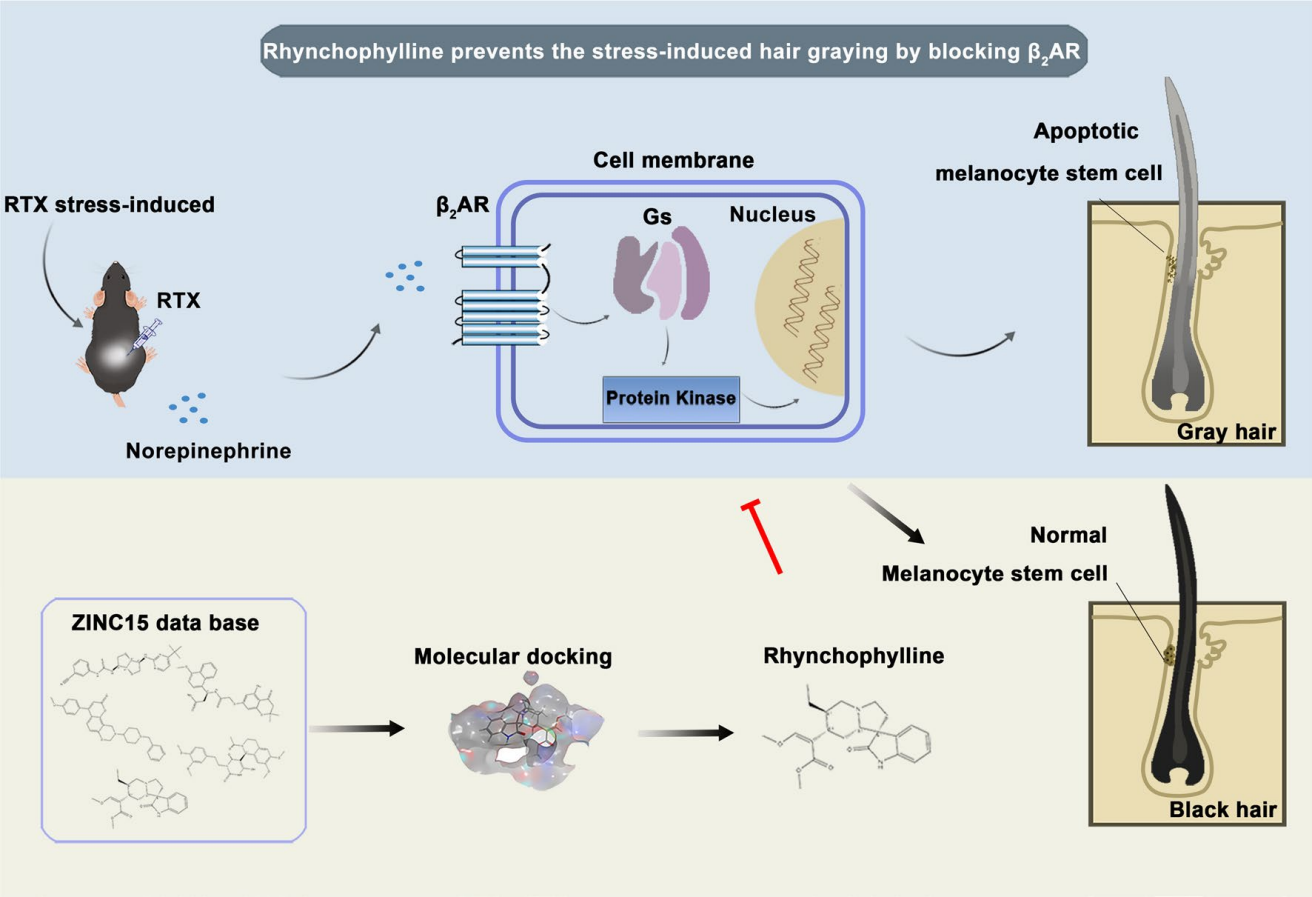

**Supplementary Information:**

The online version contains supplementary material available at 10.1007/s13659-023-00421-z.

## Introduction

Traditionally, hair graying is a normal progressive process related to the age. During this process, increasing oxidative stress [1, 2] and neuroendocrine signals [3] were the major contributors to the depletion of melanocyte stem cells (McSCs) which are responsible for the pigmentation of hair shaft. Currently, a number of drugs have been found to induce hair repigmentation by accident in the treatment of other diseases, and most of them are anti-inflammatory drugs, stimulus factors of melanogenesis or vitamins [4]. However, so far these compounds have not been conclusively shown to have a gray-hair treatment effect due to the deficiency of available research models.

Recent studies reported that stress, a psychological response to the environmental stimulus, could also drive the hair graying by stimulating sympathetic nerves to release norepinephrine (NA) signals to McSCs. Typically, NA has a short half-life and primarily functions in the circulatory system [5]. However, the systemic adverse effects of NA on tissues and organs during prolonged stress are not well understood. β_2_AR, expressed on the McSCs, has been identified as the primary receptor responsible for NA signaling [6]. Thus, blocking this receptor could be a crucial strategy for mitigating the toxic effects of NA on McSCs. Moreover, an in-depth exploration of the downstream impacts of NA on this receptor can significantly enhance the broader applicability of stress-induced gray hair models for more precise drug screening.

β_2_AR signaling has long been a model system for studying the inner workings of the G protein-coupled receptors (GPCR) family [7, 8]. As an archetypal GPCRs, β_2_AR is involved in a majority of physiological functions under the binding of hormones and neurotransmitters [9–11]. At present, many small molecule agonists of β_2_AR have been used in the clinical treatment of diverse disease, such as bronchospasm asthma and chronic obstructive pulmonary disease, while few selective antagonists were developed [12]. Propranolol is a widely used non-selective β adrenergic receptor inhibitors which block the NA effect by binding both β_1_AR and β_2_AR [13]. However, it has been used less frequently in some countries because of the carrying risks to impair glucose recovery in insulin-dependent diabetes mellitus [14]. With the discovery of its function on McSCs, there is an urgent need to develop specific blockers for this target to prevent the stress-induced hair graying. Given the fact that traditional drug development process is extremely tedious and difficult, developing the natural blockers of β_2_AR to cut off the NA transduction on McSCs maybe the most viable way for subsequent application in hair graying.

Therefore, in the present study, we used computer-aided drug design (CADD) and ZINC15 database for high-throughput screening of β_2_AR inhibitors, especially the natural inhibitors. Molecular docking is used to predict the best binding mode of the ligands to target proteins by scoring different conformations within the binding site [15]. Molecular dynamics simulations are routinely further applied to refine the obtained docking results by analyzing the intermolecular interactions and the complex stability under perturbations in an atomic level and temporal resolution [16–19]. This will provide the dynamic view of the interaction system and the atomic-level structural basis for designing and optimizing the biomolecules as well as deciphering the functional mechanisms. The obtained molecules were proceeded to the functional verification at the cell and animal level. And, an in-depth mechanism underlying the toxicity of NA to McSCs was further investigated. We found that rhynchophylline exhibited a remarkable inhibitory effect on NA-β_2_AR signaling transduction and obvious prevention effect on hair graying.

## Results

### Molecular docking studies

**Fig. 1:**
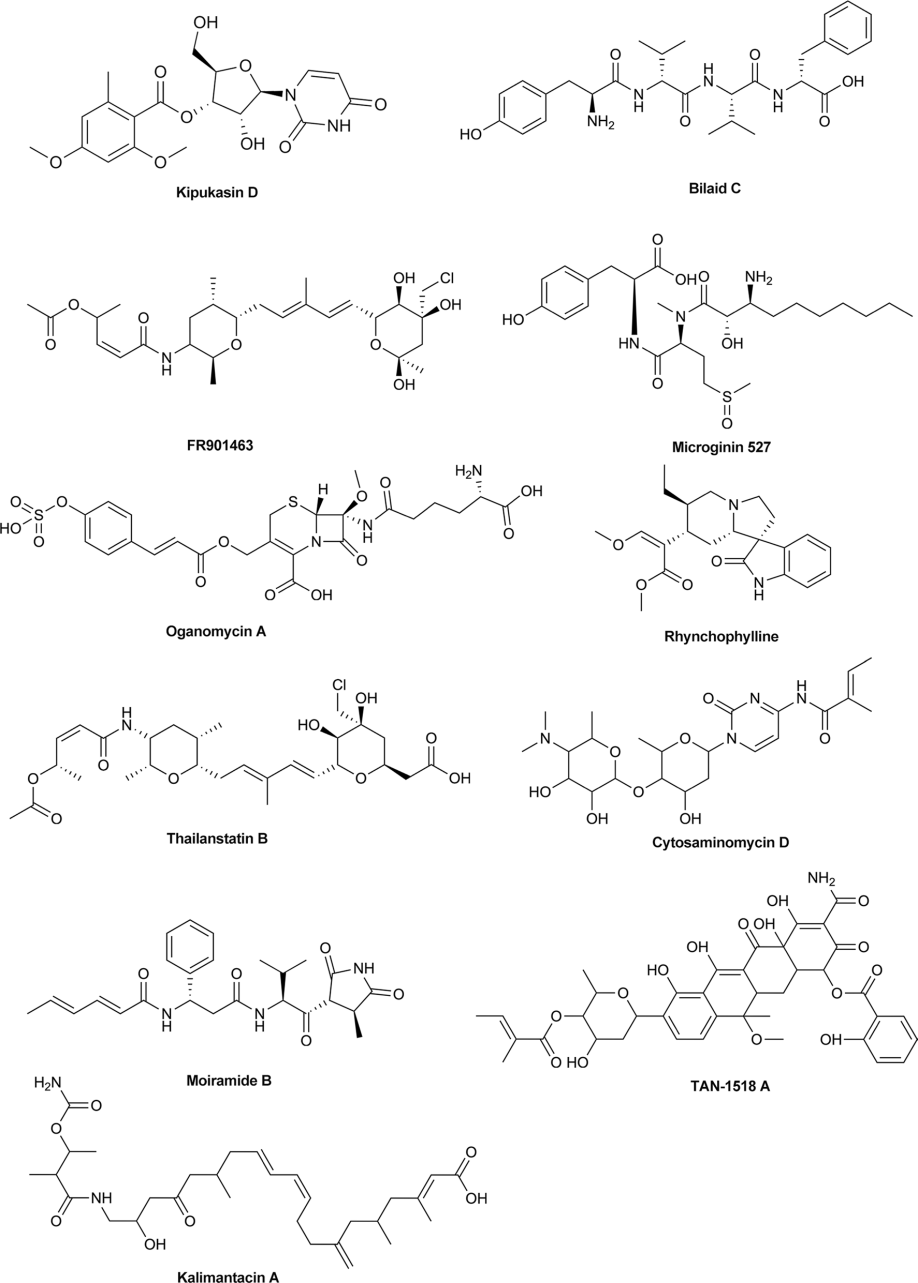
Chemical structures of the eleven selected natural compounds

**Fig. 2:**
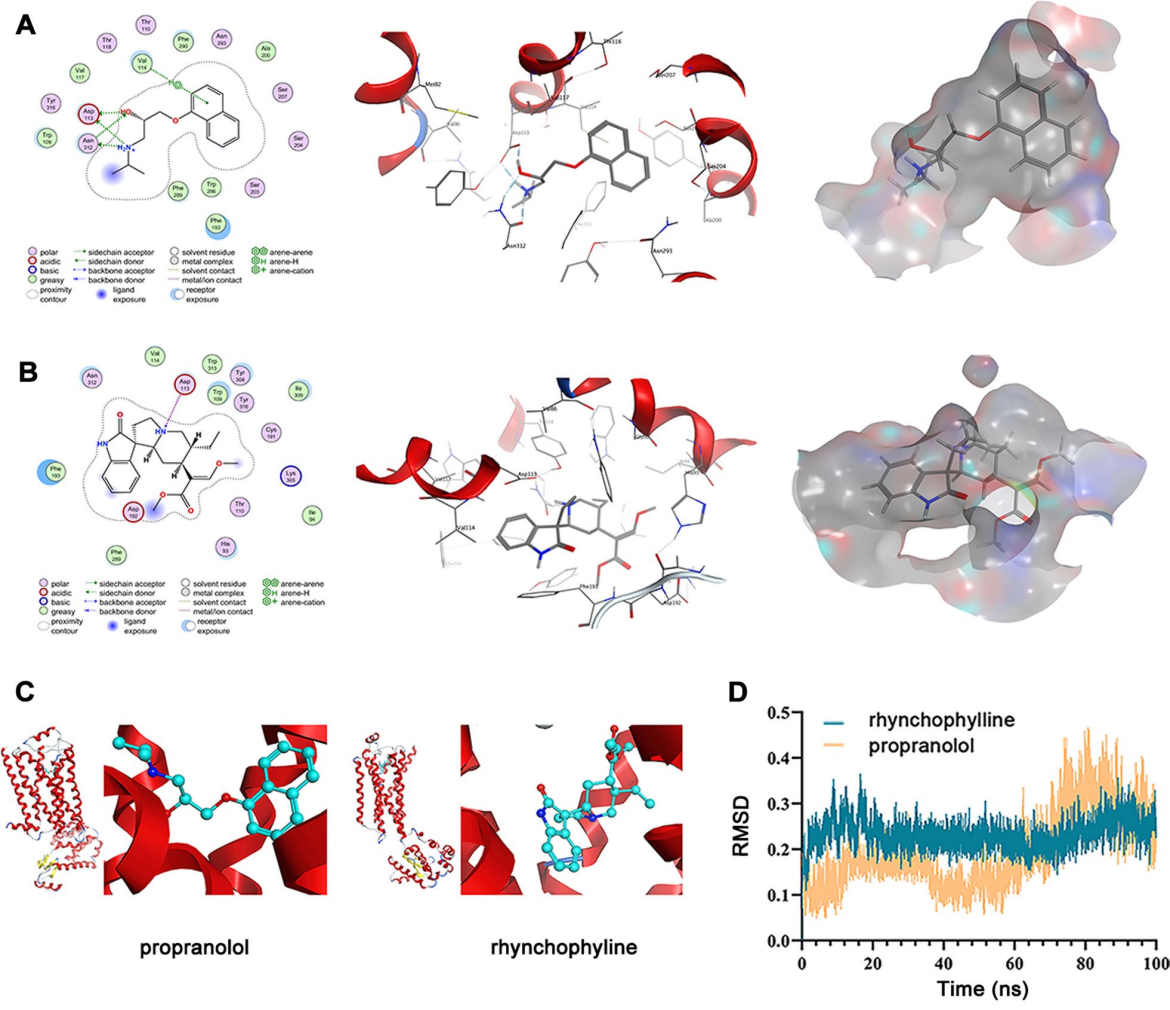
Docking parameters of the candidate molecules. **A** Crystal structures of propranolol within the active pocket of β_2_AR; **B** Crystal structures of the selected natural compound rhynchophylline within the active pocket of β_2_AR; **C** Structure diagram of β_2_AR binding to propranolol and rhynchophylline; **D** RMSD backbone of the ligand-β_2_AR complexes throughout 100 ns molecular dynamics simulation

As demonstrated, β_2_AR was the receptor of hormone NA which was the major contributor of stress signal transduction. Hence, blocking β_2_AR might be the key to prevent stress-induced hair graying. In this study, molecular docking experiment was conducted using the MOE software to screen inhibitors of β_2_AR in ZINC15 database and the space of interest was defined on the natural products. Ultimately, 11 promising natural compounds with excellent binding activity to the β_2_AR were obtained and shown in Fig. 1. It's worth noting, however, that most of these compounds turned out to be antibiotics, such as Kalimantacin A, Moiramide B, and TAN-1518 A [20–22]. Some of the remaining compounds have been investigated in anti-tumor studies, such as FR901463 [23]. Rhynchophylline attracted our attention as a cardiovascular and neuro-related drug [24, 25]. Propranolol, a known β adrenergic receptor blocker approved by US FDA, was as a positive control to be docked with β_2_AR and hydrogen-bonding interactions were seen with the resides Val114, Asp113, and Asn312 (Fig. 2A). Similarly, rhynchophylline also exhibited well docking results within the β_2_AR active site and hydrogen-bonding interactions with residue Asp113, indicating a strong affinity against to the receptor (Fig. 2B).

### Molecular dynamics simulation

To investigate the ligand-receptor stability within the binding site in a natural situation, 100 ns molecular dynamics simulations of propranolol-β_2_AR and rhynchophylline-β_2_AR complex were carried out. After accomplishment, RMSD of these two complexes were determined and the backbone values were presented in Fig. 2D. In terms of the entire simulation process, propranolol-β_2_AR complex depicted a lower average RMSD values (0.194 ± 0.082 Å) than rhynchophylline-β_2_AR complex (0.232 ± 0.032 Å). However, an obvious fluctuation of propranolol-β_2_AR appeared around 60 ns, while rhynchophylline-β_2_AR trajectories maintained a steady trend till the end of the simulation. These results demonstrated a more stable binding with β_2_AR’ active site of rhynchophylline than propranolol, which also suggested that it may be a potent inhibitor of β_2_AR.

### Natural product rhynchophylline is a potent inhibitor of β_2_AR

**Fig. 3:**
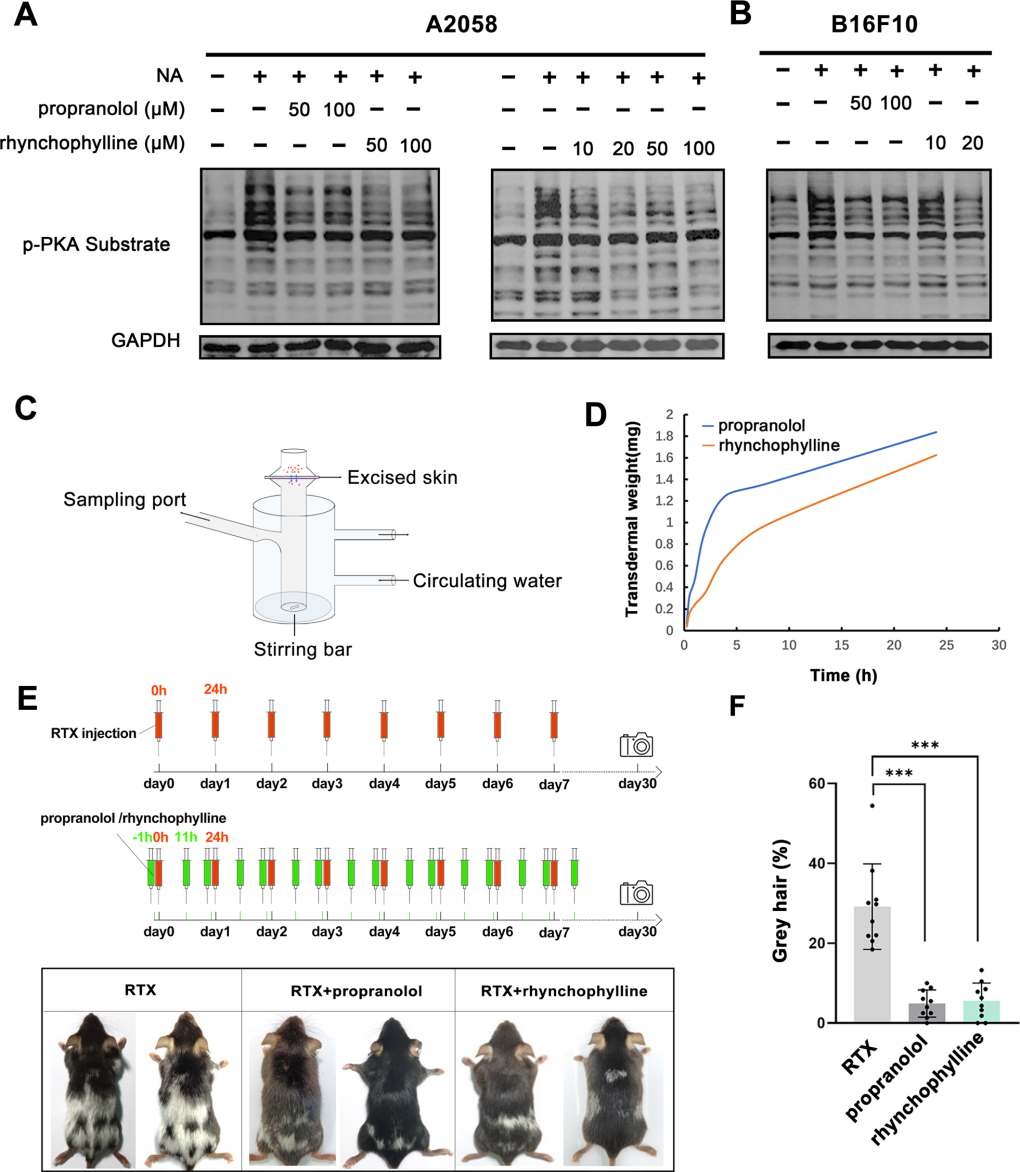
Rhynchophylline prevented the stress-induced hair graying by blocking β_2_AR signaling pathway. **A**, **B** A2058 and B16F10 cells treated with different concentrations of propranolol or rhynchophylline were subjected to western blot with anti-p-PKA substrate antibody; **C** Schematic diagram of the Franz cells; **D** Transdermal permeation efficiency of propranolol or rhynchophylline at a series of time points by measuring the concentrations in the acceptor region; **E** The mice were treated with different compounds for 7 days and then photographed at day 30; **F** The hair-graying ratios of different treated groups measured and calculated by ImageJ software. RTX induced stress mouse model was used as control group, and the control group was compared with the two experimental groups (propranolol or rhynchophylline) respectively. *** means P < 0.001 (n = 10 mice for each group, one-way ANOVA with Tukey’s multiple comparisons), the error bar is standard deviation

To verify whether the melanoma cell lines A2058 and B16F10 are suitable as cell models for testing the inhibition efficiency of rhynchophylline in blocking β_2_AR, RNA sequencing data of these two cells were obtained from NCBI database (https://www.ncbi.nlm.nih.gov/) and the expression profile of all adrenergic receptors was evaluated. The data suggested that ADRB2 and Adrb2 genes which encode the β_2_AR in human and mouse, respectively, was the predominant β androgen receptor in the melanoma cells (Additional file 1: Fig. S1A, B). In addition, these two cells are classical cell models for studying melanogenesis [26]. Therefore, the following assays involved with the inhibition effect of rhynchophylline on β_2_AR signaling were all carried out on A2058 and B16F10 cells. Given β_2_AR is the receptor of NA and transmit this signal by activating the downstream PKA, the blocking effect of β_2_AR inhibitors could been determined by detecting the level of PKA substrate phosphorylation under the NA treatment. Compared to the NA-only treated group, an obvious reduction in phosphorylated (p) PKA substrate was observed in A2058 cells treated with propranolol and rhynchophylline (Fig. 3A, B). In order to further investigate the β_2_AR inhibit efficiency of rhynchophylline, a series of concentrations rhynchophylline were treated on A2058 cells. The result showed that rhynchophylline exhibited a significant inhibition effect at 10 µM, which also occurs in B16F10 cells (Fig. 3A, B). These results suggested rhynchophylline could effectively inhibit the NA signal transduction by blocking β_2_AR.

### Rhynchophylline prevented the stress-induced hair graying

To test the transdermal properties of rhynchophylline, the in vitro skin permeation experiments were performed using the mice skin as previously reported [27, 28]. Compared with propranolol, rhynchophylline possesses similar transdermal capability with apparent transdermal weight and penetration rate in the prescribed time (Fig. 3D). Then, Propranolol and rhynchophylline were topically applied on the RTX-induced stress mouse models to elucidate their hair graying prevention effect. The results showed that gray hair was widely distributed on the back of mice injected only with RTX, indicating the successful establishment of hair graying models. Notably, less gray hair was observed after the treatment with propranolol and rhynchophylline (Fig. 3E). For a more intuitive view of the results, the mice were all photographed, and gray hair ratios on the overall back area were measured and calculated by the ImageJ software. The result showed that gray hair areas on the RTX-treated control mice was 28.5%, while with the treatment of propranolol and rhynchophylline, gray hair areas were significantly reduced to 7.5% and 8.2%, respectively (Fig. 3F). We further analyzed the single cell transcriptome data from previous studies and confirmed β_2_AR are dominantly expressed on melanocytes, in both mice and human skin. The expression pattern was consistent with that of above melanoma cells (Additional file 1: Fig. S1C, D). These data supported that β_2_AR is an important target to inhibit stress induced hair graying, and rhynchophylline is an efficient natural β_2_AR blocker and a potential compound in preventing stress induced hair graying.

### Gene expression pattern of treated cells

To investigate the mechanism underlying the preventive effect of rhynchophylline on stress-induced hair graying, RNA-seq analysis of A2058 cells was performed. The cells were treated with different concentrations of NA alone or with rhynchophylline. DMSO-treated cells were utilized as the control, with three independent replicate samples for each group. The RNA-seq data was submitted to GSA for Human at the China National Center for Bioinformation (CNCB) and assigned the accession number HRA005531.

After data cleanup and normalization, Principal Component Analysis (PCA) was performed to evaluate the replicability within each group and the differences between groups (Additional file 1: Fig. S2A). The obtained results depicted a strong resemblance among samples within the same group, while also revealed noticeable variations between different groups. Furthermore, differentially expressed genes (DEGs) were analyzed between each pair of groups. In the NA-treated groups, a total of 413 DEGs at a concentration of 100 µM and 709 DEGs at 500 µM were identified, respectively, compared to the control samples. Under the blocking effect of rhynchophylline, 556 and 534 genes underwent changes, respectively (Additional file 1: Fig. S2B).

### Rhynchophylline blocked the down-regulation of genes related with melanogenesis mediated by NA

**Fig. 4:**
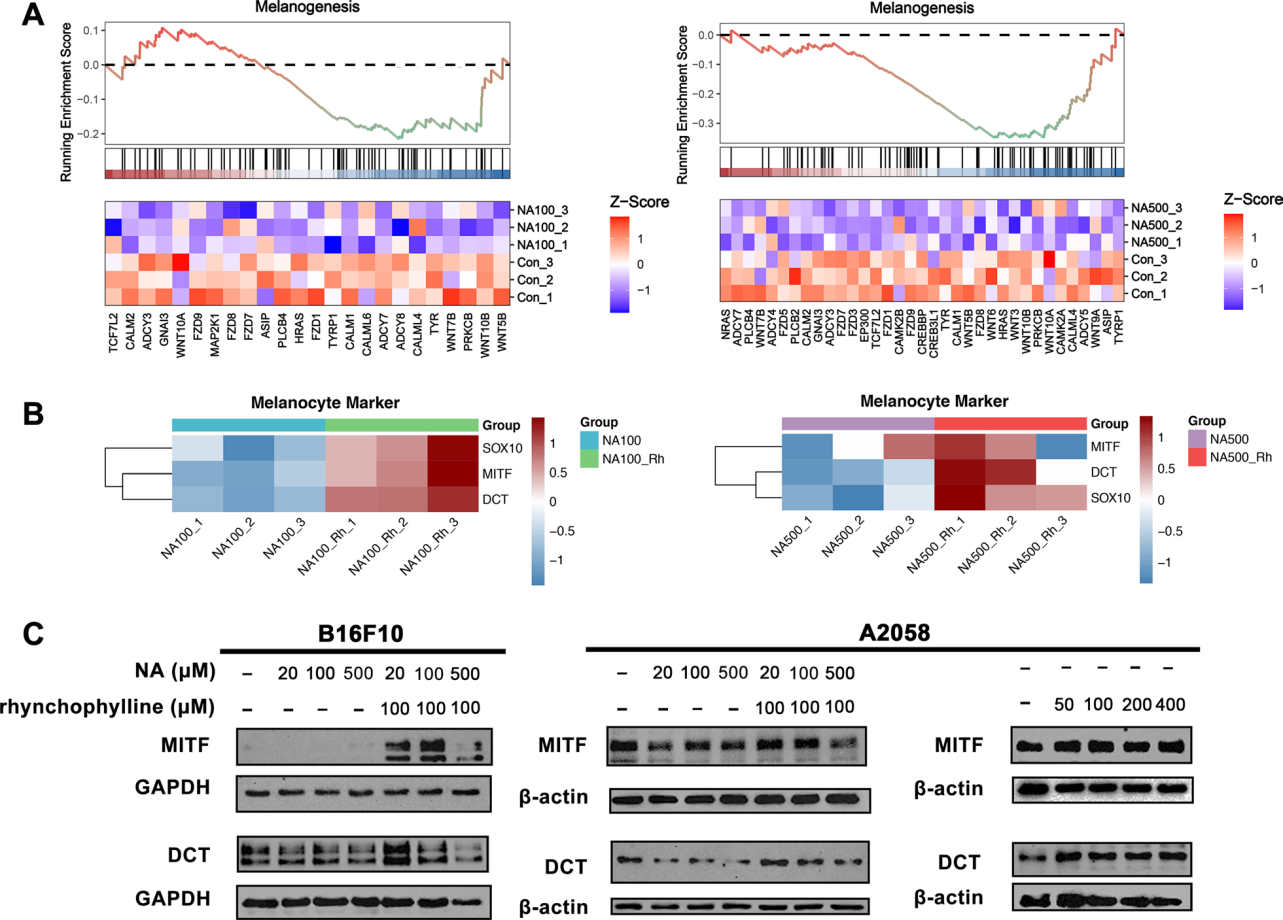
The impact of NA and rhynchophylline on melanogenesis. **A** GSEA of melanogenesis in NA-treated A2058 cells; **B** Relative expression level of melanocyte stem cells related genes in NA alone or NA-rhynchophylline treated A2058 cells; **C** A2058 and B16F10 cells treated with different concentrations of NA and rhynchophylline were subjected to western blot with anti-MITF and anti-DCT antibody. NA100: 100 µM NA treated; NA500: 500 µM NA treated; NA100-Rh: 100 µM NA and 100 µM rhynchophylline treated; NA500-Rh: 500 µM NA and 100 µM rhynchophylline treated

GSEA was proceeded to investigate the changes in melanogenesis of the treated cells (Fig. 4A). In both concentrations of NA-treated cells, a significant down-regulation of the melanogenesis pathway was observed (Fig. 4A). Therefore, in order to determine whether rhynchophylline could block this effect of NA and effectively restore the melanogenesis, the expression level of melanocyte stem cells related genes, such as SOX10, DCT, and MITF, was analyzed. The data showed that these genes were obviously upregulated in the presence of rhynchophylline, indicating a blocking effect on NA (Fig. 4B), which aligned with previous findings on mice (Fig. 3E).

Following this, the expression of MITF and DCT at the protein level was also examined. The results observed in mouse and human cell lines showed slight differences. In B16F10 cells without any external stimuli or with NA treatments, the expression of these two proteins were nearly absent. However, upon the addition of rhynchophylline, a significant increase in protein expression was observed (Fig. 4C). In A2058 cells, the results demonstrated that the expression of these genes was suppressed by NA treatment, indicating a negative impact on melanocyte stem cells (Fig. 4C). However, in the presence of rhynchophylline, the expression was restored (Fig. 4C). Based on these observations, the expression level of A2058 cells treated with rhynchophylline only was examined. We were surprised to discover that treatment with the rhynchophylline alone also enhances MITF and DCT’s expression. Collectively, these results provided additional supporting evidence that rhynchophylline could preserve the functionality of melanocyte stem cells via not only counteracting the inhibitory effects of NA, but also enhancing the genes activity itself, thereby offering a potential preventive measure against hair graying.

### Rhynchophylline prevented NA-induced Ca^2+^ influx

**Fig. 5:**
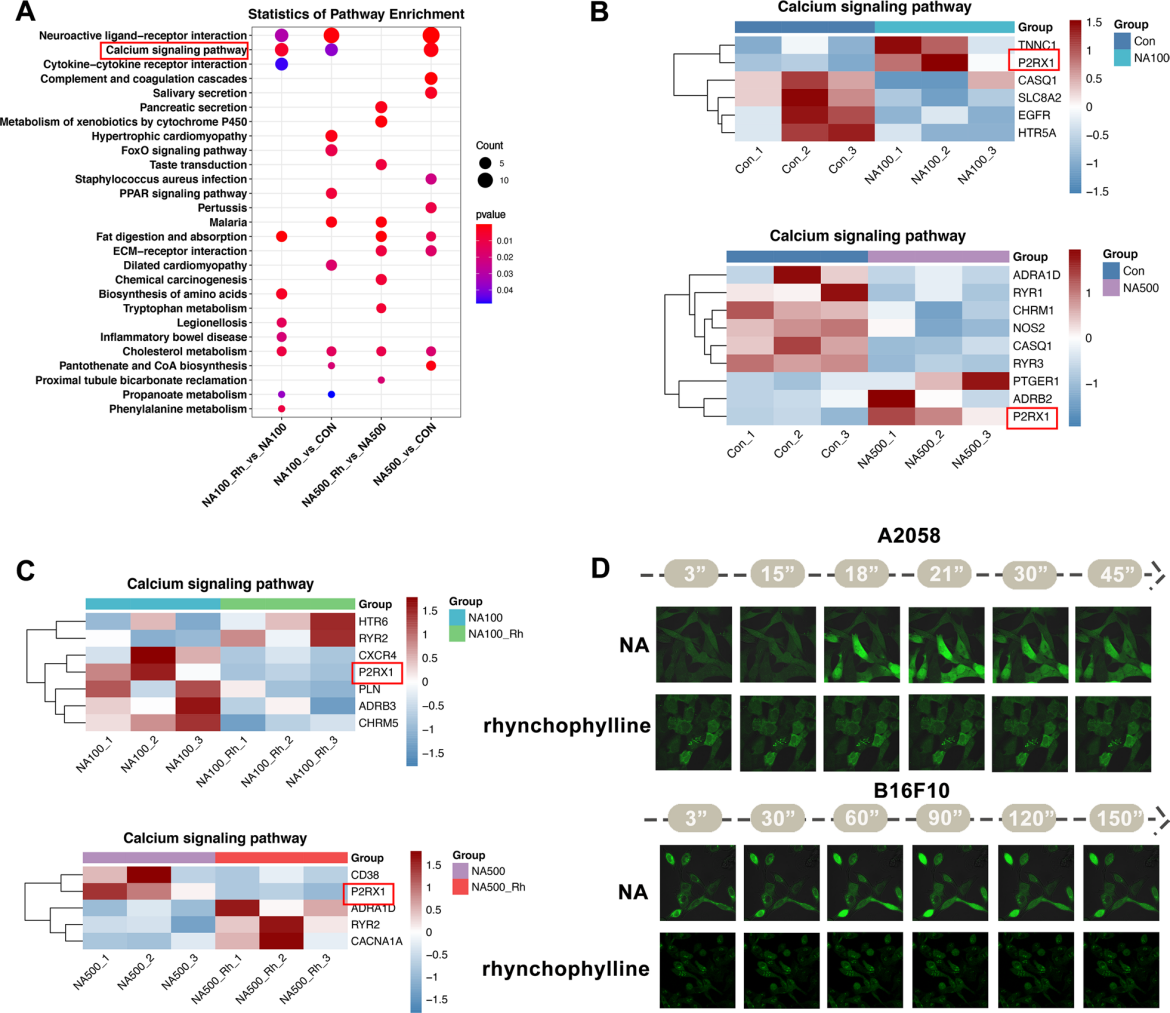
The impact of NA and rhynchophylline on calcium signaling pathway. **A** KEGG enriched pathways in NA alone or NA-rhynchophylline treated A2058 cells; **B** Relative expression level of a list of genes involved in the calcium signaling pathway in NA treated A2058 cells; **C** Relative expression level of a list of genes involved in the calcium signaling pathway in the NA alone or NA-rhynchophylline treated A2058 cells; **D** Calcium signals’ alteration within the A2058 and B16F100 cells after the NA alone or NA-rhynchophylline treatment. NA100: 100 µM NA treated; NA500: 500 µM NA treated; NA100-Rh: 100 µM NA and 100 µM rhynchophylline treated; NA500-Rh: 500 µM NA and 100 µM rhynchophylline treated

**Fig. 6:**
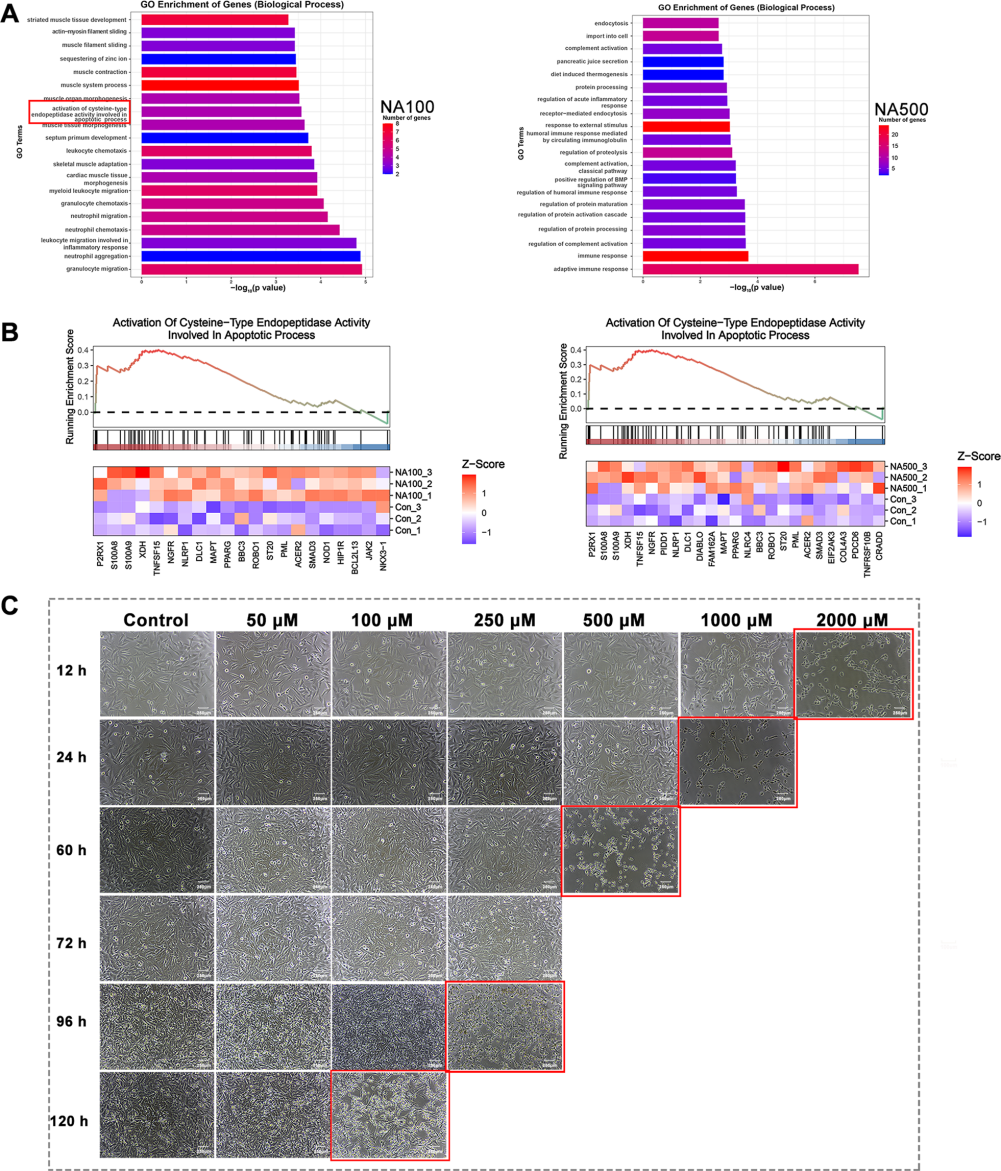
NA-induced apoptosis of A2058 cells. **A** Go term enrichment pathways of the up-regulated DEGs of A2058 cells treated with NA; **B** GSEA results of the apoptotic pathway in NA-treated A2058 cells; **C** Cell morphological observations of A2058 cells under the treatment of different concentrations of NA, the red box shows the apoptotic period. NA100: 100 µM NA treated; NA500: 500 µM NA treated. Scale bars: 250 µm

To elucidate the mechanisms influencing melanogenesis, we conducted KEGG pathway and GO function enrichment analyses on the NA only or with rhynchophylline treated groups (Figs. 5 and 6). The KEGG enrichment analysis revealed that the calcium signaling pathway was predominantly involved in the treated groups (Fig. 5A). Consequently, a list of genes associated with this pathway was examined. The results showed that NA altered the transcriptional expression of these genes compared to the control group (Fig. 5B). However, when rhynchophylline was added, the expression was turned back to a pattern similar to the control (Fig. 5C). Among them, the expression of P2RX1, which has been previously known to mediate rapid and selective permeability to cations, displayed a notably trend [29]. This suggested that rhynchophylline effectively blocked the NA-induced pathway.

Afterwards, we proceeded to investigate calcium signals within the A2058 and B16F10 cells by utilizing the Fluo-4 AM fluorescent calcium probe kit. The results demonstrated a notable increase in fluorescence intensity when the cells were treated with NA alone, indicating the possibility of calcium ion (Ca^2+^) influx occurring within the cells upon NA stimulation (Fig. 5D). However, in the group where rhynchophylline was added in addition to NA, this phenomenon was absent, which supported the previous findings regarding the blocking efficiency of rhynchophylline on the NA-induced pathway and its impact on calcium signaling in A2058 and B16F10 cells.

### Rhynchophylline prevented NA-induced cell apoptosis

**Fig. 7:**
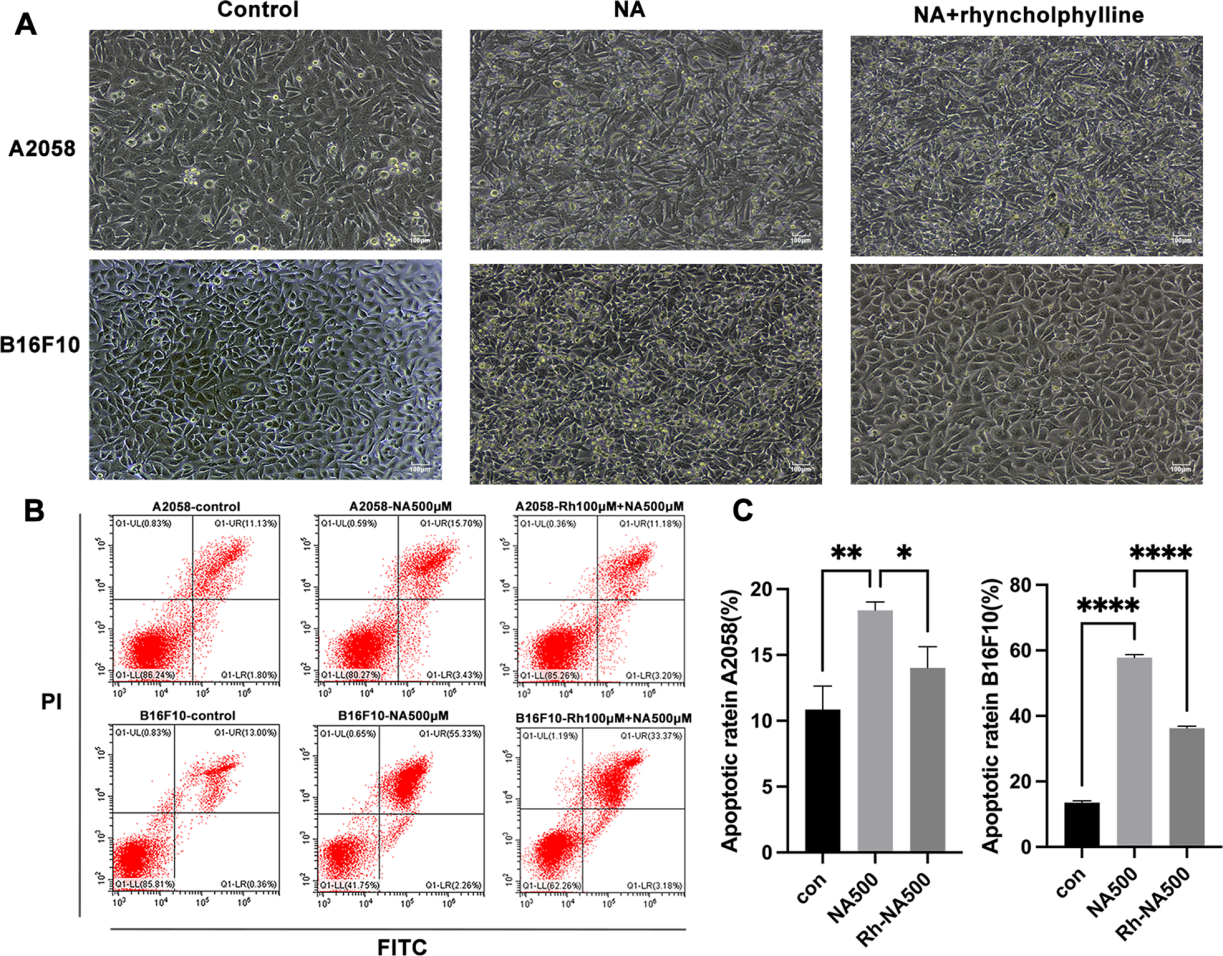
The impact of NA and rhynchophylline on cell activity. **A** Cell morphological changes of NA-only or in combination with rhynchophylline treated A2058 and B16F10 cells for 48 h; **B** Cell apoptotic rate of A2058 treated with 500 µM NA alone or in combination with rhynchophylline (Rh-NA) for 48 h, the apoptotic rate of B16F10 cells was measured after 96 h of exposure to the same treatments; **C** The cell apoptosis ratios of different treated groups were analyzed using GraphPad Prism 9. Scale bars: 100 µm. Statistical significance was set at a p-value (* < 0.05, ** < 0.01, *** < 0.001, **** < 0.0001)

In addition to its role in calcium signal mediation, P2RX1 is implicated in various cellular processes, including cell death [30]. Based on the above results that NA induced the Ca^2+^ influx, we hypothesized that NA treatment could disrupt cell activity by disturbing the calcium homeostasis within the cell, thereby influencing melanogenesis. To test this hypothesis, we performed Go term analysis on the DEGs that were upregulated. The results revealed a significant upregulation of the apoptotic pathway upon treatment with 100 µM NA (Fig. 6A, B). At higher concentrations, inflammatory pathways became more prominent, potentially due to acute cell death (Fig. 6A). To further investigate the potential induction of cell apoptosis by NA and the ability of rhynchophylline to mitigate cell death, we treated A2058 cells with various concentrations of NA and made daily observations to monitor cell behavior. Cells treated with higher concentrations of NA, such as 1000 µM and 2000 µM, exhibited rapid cell death within a day. Even at a lower concentration of 100 µM, gradual changes in cell morphology were observed, ultimately leading to cell death (Fig. 6C). However, when rhynchophylline was added to the treatment, the morphological changes in both A2058 and B16F10 cells were attenuated, suggesting a potential inhibitory effect on NA (Fig. 7A). Further investigation was conducted on B16F10 cells under the same conditions, and it was observed that the morphological changes were relatively less pronounced compared to A2058 cells. To explore this observation, the duration of drug treatment on B16F10 cells was extended to 96 h for the subsequent experiment (Fig. 7A).

In continuation of the previous findings, we conducted a cell apoptotic assay using the Annexin V kit to further investigate the rates of cell death. The results illustrated that treatment with NA induced significant apoptosis rates in both A2058 and B16F10 cells. Specifically, A2058 cells displayed an apoptosis rate of 20.42%, while B16F10 cells exhibited a higher rate of 58.50% (Fig. 7B, C). However, in the presence of rhynchophylline, the apoptosis rate was reduced to 16.5% and 37.49%, respectively. These results further support our earlier observations and emphasize the protective effect of rhynchophylline against NA-induced apoptosis in both cell lines.

Based on these findings, it is reasonable to hypothesize that prolonged and chronic exposure to stress could lead to the apoptosis of vulnerable melanin stem cells due to sustained expression of NA. This sustained cellular stress and ensuing apoptosis may contribute to the onset of gray hair. Further investigation is needed to elucidate the underlying mechanisms regulating melanogenesis. Nonetheless, our results suggested that modulating calcium signaling and protecting against apoptosis could be potential strategies for preventing or mitigating hair graying.

## Discussion

In this study, our original objective was to identify effective inhibitors for β_2_AR, with the goal of disrupting the receptor's interaction with NA, which would prevent the onset of hair graying. In this particular section, CADD techniques and ZINC15 database were utilized for a high-throughput screening of natural β_2_AR inhibitors. After rigorous molecular docking evaluations, one particular natural product piqued our interest—rhynchophylline.

Rhynchophylline is naturally found in various species of *Uncaria* and has a rich history of use in different regions, including Southeast Asia and Africa. In China, rhynchophylline obtained from *Uncaria macrophylla, Uncaria sinensis, Uncaria sessilifructus*, and *Uncaria rhynchophylla* was included in the Chinese Pharmacopoeia (China, 2005) and has been widely utilized in traditional Chinese medicine. Its applications have ranged from alleviating wind-related ailments, managing convulsions, clearing heat, to soothing liver-related conditions [31]. Furthermore, it has been employed in the treatment of central nervous and cardiovascular diseases, which is the point that catches our attention the most. Another remarkable aspect of rhynchophylline is its excellent safety profile. It is even listed in the Catalog Used of Cosmetics Raw Materials (China, 2021), which opens up multiple avenues for its development. Being a natural product, rhynchophylline can potentially find its way into clinical applications more swiftly than synthetic chemical compounds. Therefore, we aimed to further investigate the underlying mechanisms of stress on hair graying and to determine whether rhynchophylline could effectively block these additional downstream pathways of NA-mediated.

In the process of further exploring the mechanism, we found that NA could influence the melanogenesis, the calcium homeostasis and the cell activity. Melanogenesis is a multifaceted process encompassing distinct stages, including a series of enzymatic reactions and molecular events within these cells, resulting in the production and distribution of melanin granules throughout various tissues in the body. In this pathway, cAMP acts as a crucial role in the regulation [32]. It initiates a signaling cascade that involves the activation of PKA, which then phosphorylates CREB. Phosphorylated CREB enhances the transcription of MITF, which plays a central role in melanocyte development and melanin synthesis [33, 34]. This interconnected regulatory network ensures the precise control of melanin production in melanocytes, influencing skin, hair, and eye color.

Therefore, in previous pathway studies, cAMP and MITF were found to be positively correlated. However, in our research, we observed that treatment with NA resulted in the downregulation of gene expression (MITF and DCT) related to melanogenesis, despite the activation of the cAMP pathway. Consequently, we hypothesized that the relationship between the melanogenesis pathway and the cAMP pathway is subject to multifaceted signaling regulation, and the treatment with NA may have triggered other signaling pathways, potentially affecting this connection, depending on the timing and concentration of NA exposure. Therefore, we conducted further analysis and discovered alterations in calcium signaling and apoptotic signaling in NA-treated cells.

Calcium (Ca^2+^) serves as important second messengers in various cellular signaling pathways, including those involved in melanogenesis [35]. For example, changes in intracellular calcium levels can trigger a cascade of events that affect the activity of transcription factors like MITF. It is noteworthy that calcium signaling is intricately interconnected with an array of other signaling pathways, notably including cAMP signaling, as previously highlighted [36]. The existence of cross-talk between these pathways serves as an elegant mechanism for fine-tuning the regulation of melanogenesis in response to a myriad of physiological and environmental cues.

In addition, a profound correlation exists between calcium signaling and apoptosis, the meticulously orchestrated process of programmed cell death. Changes in intracellular calcium concentrations can trigger a series of signaling pathways, some of which are directly related to apoptosis. For example, elevated intracellular calcium levels can affect the permeability of the mitochondrial membrane, leading to a loss of mitochondrial membrane potential and the release of apoptotic factors, such as cytochrome C, which initiates the apoptotic cascade. Calcium signaling can also modulate the activity of proteins directly involved in apoptosis, such as caspases [37]. In addition, calcium signaling can interact with other signaling pathways, such as death receptor signaling, to regulate apoptosis [38]. This interaction can affect the sensitivity of cells to apoptotic signals and their responsiveness to apoptosis-inducing stimuli.

As mentioned above, in our study, NA treated cells showed changes in calcium signals, cell apoptosis, and down-regulation of melanin synthesis, which were prevented after the addition of Rhynchophylline. Therefore, we proposed a hypothesis that NA affects calcium homeostasis by regulating cAMP pathway, which further leads to cell apoptosis and affects melanin production. However, the deeper connection between these pathways needs to be further explored.

## Conclusion

All together, these results demonstrated that rhynchophylline is an efficient blocker of β_2_AR. Therefore, we conducted the in vivo experiments on the RTX-induced stress mice, obviously less area of hair graying was found compared to the untreated mice, indicating its prevention effect. In addition, RNA-seq data was further suggested that rhynchophylline could preserve the melanogenesis capacity by blocking the NA-induced calcium alterations and cell apoptosis. These new findings are of tremendous value and provide new insight for the other types gray hair studies and the further application.

## Materials and methods

### Molecular docking

The docking studies were performed using Molecular Operating Environment software (MOE, 2015.10) and the detailed procedure was described earlier [39]. Briefly, the three-dimensional structure of the target β_2_AR was downloaded from RCSB PDB (6PS5) and further modified by removing the heteroatoms and water molecules in the MOE software. 642129 molecules to be tested were collected from ZINC 15 database and transferred to MOE to carry out the docking within the β_2_AR’s active site. In addition, propranolol, a known β adrenergic receptor inhibitor, was selected as a positive control. Its structure was obtained from ChemicalBook (www.chemicalbook.com). The binding effect of the compounds with β_2_AR was evaluated through their binding scores. Based on the docking results, the promising natural inhibitor of β_2_AR were further selected to proceed molecular dynamics simulation.

### Molecular dynamics simulation

For understanding the structural-basis of the function mechanism between the selected inhibitors and the protein targets, an initial coordinate for 100 nsec all-atom molecular dynamics simulation was carried out using a GROMACS 2020.6-MODIFIED software and a CHARMM36 force field. The ligand–protein complex was immersed in a cubic box with three-points (TIP3P) water and followed by the addition of counter ions to neutralize the solvated system. Then, the complex system was quickly energy minimized using the steepest descent minimization algorithm. Following this, two-staged equilibration with NVT (Number of particles, Volume and Temperature) and NPT (Number of particles, Temperature and Pressure) was conducted and ensemble for 100 psec. The equilibrated systems were proceeded to molecular dynamic run for 100 nsec under a constant number of particles at 310 K and 1 bar pressure. The results were analyzed and determined for root mean square deviation (RMSD).

### Cell culture

Human A2058 and murine B16F10 melanoma cell lines were both cultured in RPMI 1640 medium (Gibco) supplemented with 10% fetal bovine serum (FBS; BI) and 1% penicillin–streptomycin at 37°C with 5% CO_2_.

### Adrenergic receptors’ expression pattern analysis

To make sure whether the melanoma cell lines are suitable for testing the efficiency in blocking β_2_AR, adrenergic receptors’ expression pattern of these two cells were analyzed using the transcriptome data. Datasets GSE150348 of A2058 cells and datasets PRJNA505989 of B16F10 cells were obtained from Gene Expression Omnibus (GEO, http://www.ncbi.nlm.nih.gov/geo/) and Sequence Read Archive (SRA, https://www.ncbi.nlm.nih.gov/sra), respectively [40, 41]. Expression levels of genes were normalized and visualized by pheatmap R package. Besides, the single cell transcriptome datasets of GSE129611 and GSE129218 from GEO were also used to figureout the expression pattern of adrenergic receptors in human and mouse melanocytes [42, 43]. The quality control standard for each dataset was based on the original studies when available. After that, the cell cluster annotations were accomplished depending on the cell makers reported in the previous studies and visualized using the t-distributed Stochastic Neighbor Embedding (t-SNE) method. Average and log-normalized expression levels for adrenergic receptors were plotted using the DotPlot function in the Seurat R package.

### Blocking efficiency assay by western blot

To investigate the inhibitory effect of the selected natural inhibitors on NA signal transduction, A2058 and B16F10 melanoma cells were seeded in 6-well plates and incubated at 37 °C and 5% CO_2_ for 24 h. Then, the culture medium was exchanged with a fresh FBS-free medium containing different concentrations of propranolol or the selected compound rhynchophylline, and incubated at 37 °C and 5% CO_2_ for 30 min. After that, 100 µM NA (Sigma, #489350) was added for an additional incubation of 48 h. The inhibition assay was further conducted and detected using western blot.

The treated cells were lysed with a RIPA buffer (Beyotime, P0013) containing the protease (CWBIO, CW2200S) and phosphatase inhibitors (NCM, P003) on ice. Then, the protein was extracted by ultrasonic disintegration for three times and centrifugation at 4°C, 2000 g for 15 min. Followed by the extraction, the protein concentration in the supernatants was measured using a BCA Protein Assay Kit (Beyotime, P0009) according to the manufacturer’s instructions. After that, the prepared sample was separated by polyacrylamide gel electrophoresis (PAGE), transferred to polyvinylidene fluoride (PVDF) membranes, blocked with 5% skim milk powder (dilution: TBST) at room temperature for 1 h, and incubated with primary antibodies overnight at 4°C (dilution: TBST): anti-p-PKA Substrate (Cell Signaling Technology, 9624S, 1:1000), anti-GAPDH (GeneTex, GTX100118, 1:5000). On the following day, the membranes were incubated with HRP-linked secondary antibodies (Cell Signaling Technology, 7074P2, 1:1000) at room temperature for 1 h and visualized using a WesternBright™ ECL (Advansta, K-12045-D50).

### Transdermal permeation experiment

Transdermal permeation assays were conducted in the vertical Franz-type diffusion cells (DISA, Milan, Italy). In detail, the mice dorsal skin was placed between the donor and acceptor chamber of the Franz cell, and the dermal surface was in contact with the receptor compartment. 4 mL transdermal formulation of propranolol (1 mg/mL; Sigma-Aldrich, PHR1308) and rhynchophylline (1 mg/mL; YuanYe, B20453) dissolved in the solvent (1, 2-propylene glycol: absolute ethanol: peppermint ketone: ddH_2_O = 50:32:5:13) was set as the donor. 50 mL 1% PBS solution was as the acceptance media and maintained at 37°C by the constant stirring. Then, the experiments were carried out for 24 h. To evaluate the permeation efficiency, 1 mL of the acceptance solution for each test were taken at 15 min, 30 min, 1 h, 2 h, 4 h, 8 h, 24 h and measured using Ultra-micro-UV spectrophotometer (nanodrop N60).

### Prevention efficiency of hair-graying on resiniferatoxin (RTX)-induced Stress Mice

C57BL/6 female mice of 7 weeks old were housed under specific pathogen-free animal center with 12 h light–dark cycle and allowed for free access to water and diet. For the establishment of stress-induced hair graying models, RTX (AdipoGen Life Sciences, AG-CN2-0534) was prepared in the PBS containing 2% DMSO with 0.15% Tween 80 and was injected in the flank of the depilated mice at 20 µg/kg for 7 days. For the inhibition group, 200 µL 1 mg/mL propranolol or rhynchophylline were applied to the skin of mice twice a day, 1 h before and 11 h after the RTX injection, respectively. After the treatment, the hair graying area of the mice were photographed at day30 and measured using ImageJ software. All the animal experimental procedures were conducted in accordance with the internationally accepted principles for laboratory animal use and care as found in the European Community guidelines (EEC Directive of 1986; 86/609/EEC) and Use Committee of Sun Yat-sen University (approval no. SYSU-YXYSZ-20210332).

### RNA sample preparation and sequencing

The final concentrations of NA and rhynchophylline chosen for the treatment of A2058 cells for RNA sequencing were 100 µM, which was determined based on the results of above inhibition assay. Further, a higher concentration of NA with 500 µM was also conducted to determine whether rhynchophylline could counteract the effects of NA at an elevated concentration. A2058 melanoma cells were treated with different concentrations of NA alone or with rhynchophylline as described above for 48 h. To collect the cell samples for RNA extraction, the culture medium was discarded, and the cell was washed with PBS to remove residual medium. Next, 1 mL of Trizol reagent (Life Technologies) was added, and the mixture was vigorously pipetted to ensure complete contact and digestion of Trizol with the cells. The dissolved cell lysate was transferred to a new RNase-free centrifuge tube, where further pipetting was carried out until all cells were dissolved. The samples were sent to Chi Biotech (Guangzhou, China) for RNA extraction. Following this, its purity was assessed using Nanodrop by measuring the OD260/280 and OD260/230 ratios, while its integrity was evaluated using the Agilent 4200 TapeStation.

For mRNA library preparation, 1 µg of total RNA was utilized as the input material per sample. Initially, poly A-tailed eukaryotic mRNA was enriched using the Oligo (dT) magnetic beads. Subsequently, the enriched mRNA was randomly fragmented employing bivalent cations. To synthesize the first strand of cDNA, reverse transcriptase was utilized with segmented mRNA acting as a template, and random oligonucleotides being employed as primers. The second strand of cDNA was then synthesized using 2^nd^ Strand Enzymes and dNTPs. The resulting double-stranded cDNA was subjected to end repair, A-tailing addition and sequencing adapter ligation to generate cDNA fragments of around 200–300 bp in size. PCR amplification was performed, followed by additional purification steps to obtain the final library. To evaluate the quality of the prepared libraries, the Agilent 4200 TapeStation was utilized. After passing the library quality assessment, the prepared libraries were sequenced on a NovaSeq 6000 platform. During the sequencing process, paired-end reads of 150 bp in length were generated, providing valuable sequence information for subsequent analysis.

### RNA data analysis

The raw data in Fastq format obtained from sequencing were subjected to data processing using custom Perl scripts. This preprocessing step involved trimming reads containing adapters, poly-N sequences, or low-quality bases, resulting in clean reads. Subsequently, the paired-end clean reads were aligned to the reference genome database hg38. After the alignment, read counts mapped to each gene were obtained using the featureCounts (version 2.0.3) program [44]. Then, transcripts Per Kilobase of exon model per Million mapped reads (TPM) values were calculated. Before performing differential gene expression analysis, the read counts were normalized using the edgeR (version 3.18.1) program package through scaling factors [45–47]. Once the read counts were normalized, the differential expression analysis between two conditions was then carried out. The resulting p-values were adjusted using the Benjamini & Hochberg method for multiple testing. The threshold for significant differential expression was set at a corrected p-value of 0.05 and an absolute fold change of 2. Gene Ontology (GO) enrichment analysis, Kyoto Encyclopedia of Genes and Genomes (KEGG) pathway enrichment analysis, Gene Set Enrichment Analysis (GSEA) of differentially expressed genes were conducted using the clusterProfiler R package (version 4.22) with correction for gene length bias [48, 49].

### Melanogenesis assay

To make sure the effect of NA and rhynchophylline on the melanogenesis, the expression levels of melanocyte stem cells-related genes MITF and DCT were detected by analyzing the RNA-seq data and western blot: anti-MITF (Cell Signaling Technology,97800S, 1:1000), anti-DCT (Invitrogen, PA5-105275, 1:500), anti-β-actin (Cell Signaling Technology, 4970S, 1:1000), anti-GAPDH (GeneTex, GTX100118, 1:5000).

### Calcium signals detection

Aimed to investigate the effects of NA and rhynchophylline on calcium oscillations, A2058 and B16F10 cells were cultured on confocal culture dishes for 24 h. Fluo-4 AM fluorescent calcium probe (Beyotime, S1060, 2 mM) was then added and incubated with the cells for 30 min at 37°C. Following three washes with PBS, the cells were further incubated in Hanks' solution for an additional 30 min. To assess the impact of inhibitors, rhynchophylline was administered 30 min prior to NA addition. Subsequently, NA was added to the dish and the images were captured in a "time series" mode using a confocal laser scanning microscope (Zeiss, LSM880), and continuously for 300 s with 3-s intervals.

### Cell morphology observations

To investigate the impact of NA and rhynchophylline on the cell morphology, the cells were initially seeded onto a culture dish with a diameter of 35 mm at a density of 1 × 10^5^ cells/dish and sub-cultured for 24 h. Subsequently, the culture medium was replaced with fresh medium containing varying concentrations of NA and rhynchophylline. The cellular morphology was then observed and images were captured at different time points.

### Flow cytometry

An Annexin V kit (Yeasen, 40302ES60) was used for the analysis of apoptosis. A2058 and B16F10 cells treated with NA alone or with rhynchophylline were digested with trypsin without EDTA for 1 min. Following that, the cells were centrifuged at 300*g*, 4 °C for 5 min and washed with cold PBS twice. Subsequently, 1 × binding buffer was added and resuspend the cells. To perform the apoptosis assay, 5 µL of Annexin V-FITC and 10 µL of PI staining solution were further added and incubated with the resuspended cells at room temperature for 10–15 min. The prepared samples were analyzed within 1 h using a flow cytometer (Beckman, CytoFLEX).

### Statistical analysis

All data were analyzed using Graphpad Prism 9 (GraphPad Software). One-way analysis of variance (ANOVA) with a post-hoc Tukey's test was used for statistical comparison of the groups. Statistical significance was set at a p-value (* < 0.05, ** < 0.01, *** < 0.001, **** < 0.0001).

### Supplementary Information

**Additional file 1. Fig. S1.** Adrenergic receptors’ expression pattern on the cells. **Fig. S2. **Norepinephrine and rhynchophylline altered the gene expression pattern of A2058 cells. **Fig. S3.** Go term and KEGG enrichment analysis of the top 20 pathways in different concentrations of NA-only or with rhynchophylline treated groups.

## Data Availability

Publicly available datasets were analyzed in this study. Datasets GSE150348, GSE129611 and GSE129218 were available on GEO (http://www.ncbi.nlm.nih.gov/geo/). Datasets PRJNA505989 was available on SRA (https://www.ncbi.nlm.nih.gov/sra). Datasets HRA005531 are available on GSA for Human (https://ngdc.cncb.ac.cn/gsa-human/).
