# Supplementary material for: Natural product rhynchophylline prevents stress-induced hair graying by preserving melanocyte stem cells via the β2 adrenergic pathway suppression
Source: Nat Prod Bioprospect. 2023 Dec 1;13(1):54. doi: 10.1007/s13659-023-00421-z (PMC10689686; doi:10.1007/s13659-023-00421-z)
Supplement: Supplementary file 1 — Additional file 1. Fig. S1. Adrenergic receptors’ expression pattern on the cells. Fig. S2. Norepinephrine and rhynchophylline altered the gene expression pattern of A2058 cells. Fig. S3. Go term and KEGG enrichment analysis of the top 20 pathways in different concentrations of NA-only or with rhynchophylline treated groups. [file 13659_2023_421_MOESM1_ESM.docx]

**Additional file 1**

**Natural product rhynchophylline prevents stress-induced hair graying by preserving** **melanocyte stem cells via the β2 adrenergic pathway suppression**

**Xinxin Li^a,b,c^, Runlu Shi^d^, Lingchen Yan^a^, Weiwei Chu^a,b^, Ruishuang Sun^e^, Binkai Zheng^a^, Shuai Wang^a,f^, Hui Tan^c*^, Xusheng Wang^a*^, Ying Gao^a,b,g*^**

^a^School of Pharmaceutical Sciences (Shenzhen), Sun Yat-sen University, Shenzhen 518107, China

^b^Department of Anesthesiology, The First Affiliated Hospital of Bengbu Medical College, Bengbu 233004, China

^c^Center for Child Care and Mental Health, Shenzhen Children's Hospital Affiliated to Shantou University Medical College, Shenzhen 518026, China

^d^Institute of Biopharmaceutical and Health Engineering, Tsinghua Shenzhen International Graduate School, Tsinghua University, Shenzhen 518055, China

^e^Department of Plastic and Reconstructive Surgery, Guangdong Second Provincial General Hospital, Guangzhou 510317, China

^f^The Yonghe Medical Beauty Clinic Department, Guangzhou 510630, China

^g^Department of Anesthesiology, The First People’s Hospital of Foshan, Foshan 528000, China

*** Correspondence:**
gying1008@126.com, Ying Gao; [wangxsh27@mail.sysu.edu.cn](mailto:wangxsh27@mail.sysu.edu.cn), Xusheng Wang; [huitan@email.szu.edu.cn](mailto:huitan@email.szu.edu.cn), Hui Tan.


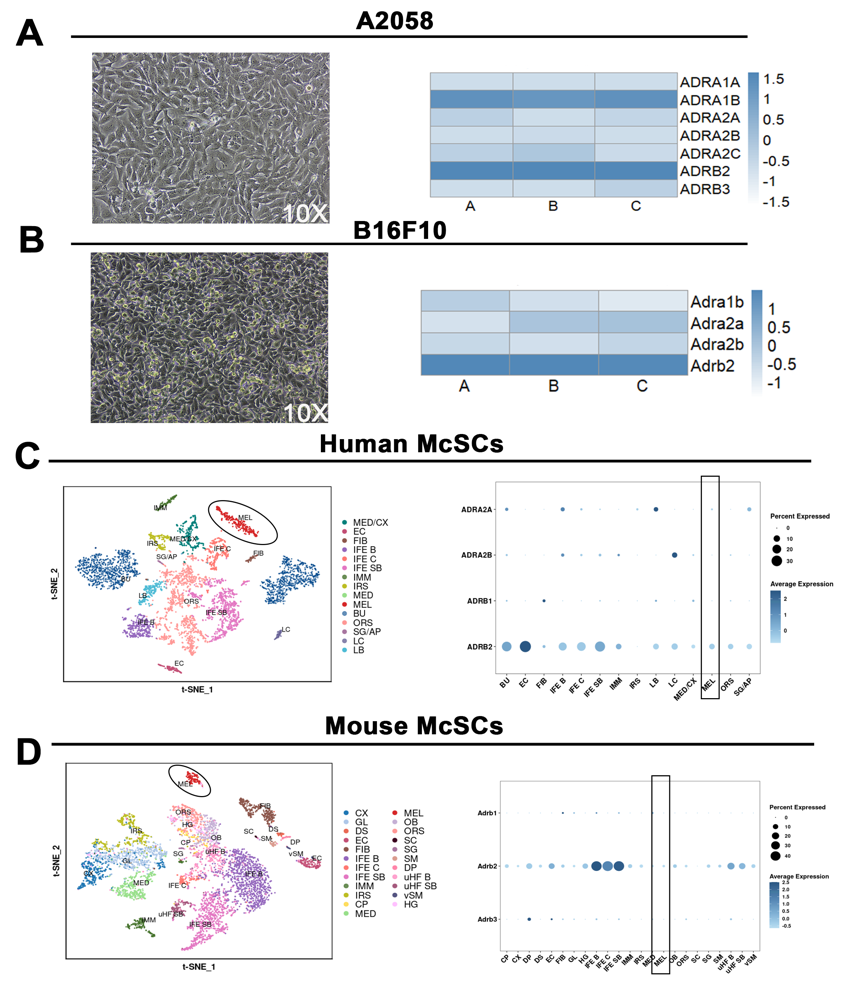


**Fig. S1** Adrenergic receptors’ expression pattern on the cells. (A) and (B) Analysis of adrenergic receptors’ expression pattern of human melanoma cell A2058 (A) and mouse melanoma cell B16F10 (B) using bulk transcriptome sequencing data; (C) and (D) Exhibition of the adrenergic receptors’ expression pattern on different human and mouse skin cell types using the single cell transcriptome sequencing data. Fibroblasts (FIB); Dermal sheath (DS:); Dermal papilla (DP); Immune cells (IMM); Endothelial cells (EC); Vascular smooth muscle (vSM); Melanocytes (MEL); Schwann cells (SC) ; Skeletal muscle (SM) ;Outer root sheath (ORS) ; Companion layer (CP); Germinative layer (GL); Inner root sheath (IRS); Cortex/cuticle (CX) ; Medulla (MED); IFE, cycling (IFE C); IFE basal (1 pop.) (IFE B); IFE suprabasal (IFE SB); Upper HF basal (uHF B); Upper HF suprabasal (uHF SB); Sebaceous gland (SG); Outer bulge (OB); Hair germ (HG); apocrine gland (AP); Bugle (BU); Lower bugle (LB); Langerhans' cells (LC).


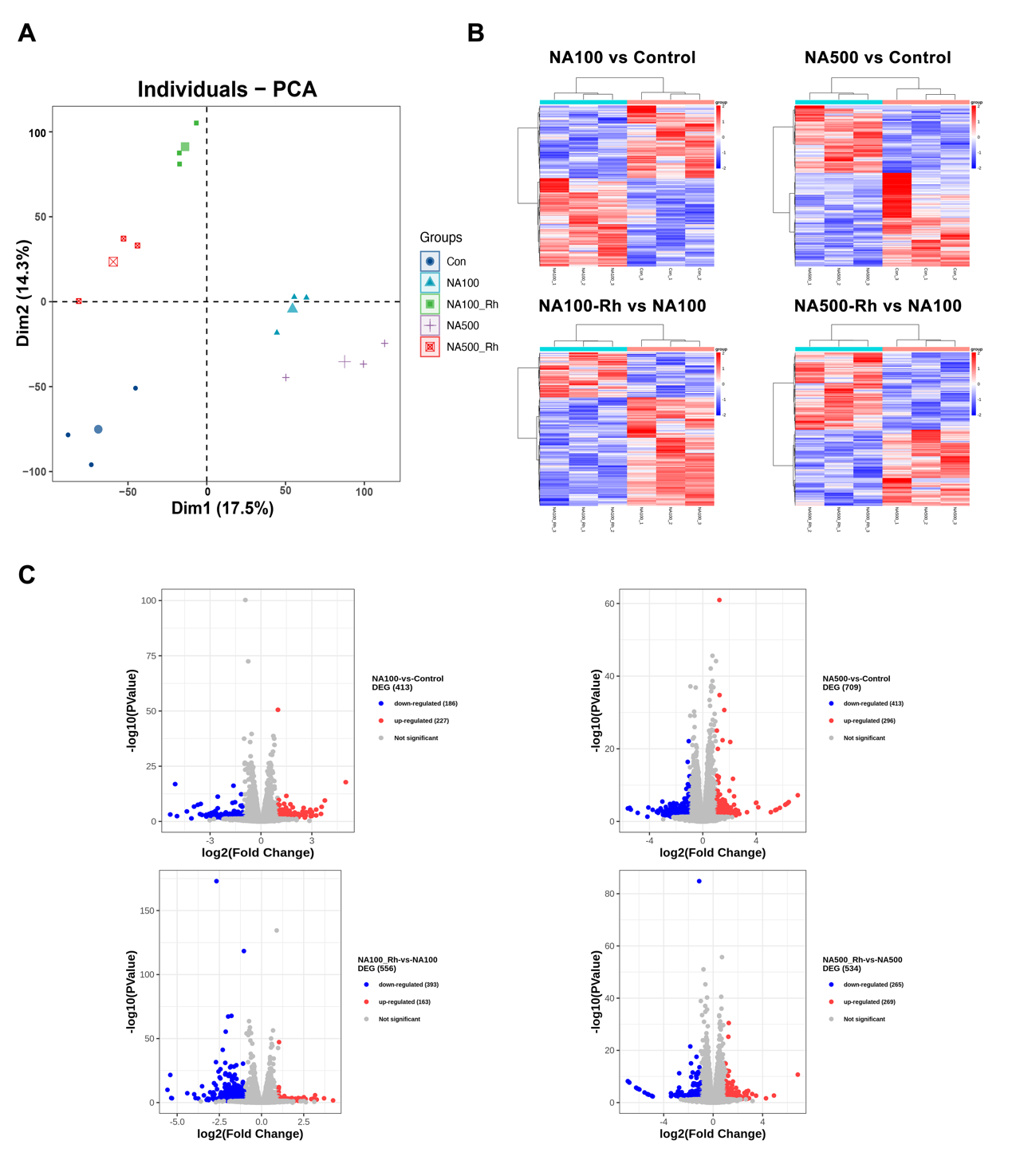


**Fig. S2** Norepinephrine and rhynchophylline altered the gene expression pattern of A2058 cells. (A) Principal Component Analysis (PCA) between treated and control groups; (B) Heat map of treated and control groups. (C) Distribution of differentially expressed genes (DEGs) between treated and control groups in the volcano plot. NA100: 100 µM NA treated; NA500: 500 µM NA treated; NA100-Rh: 100 µM NA and 100 µM rhynchophylline treated; NA500-Rh: 500 µM NA and 100 µM rhynchophylline treated.

**
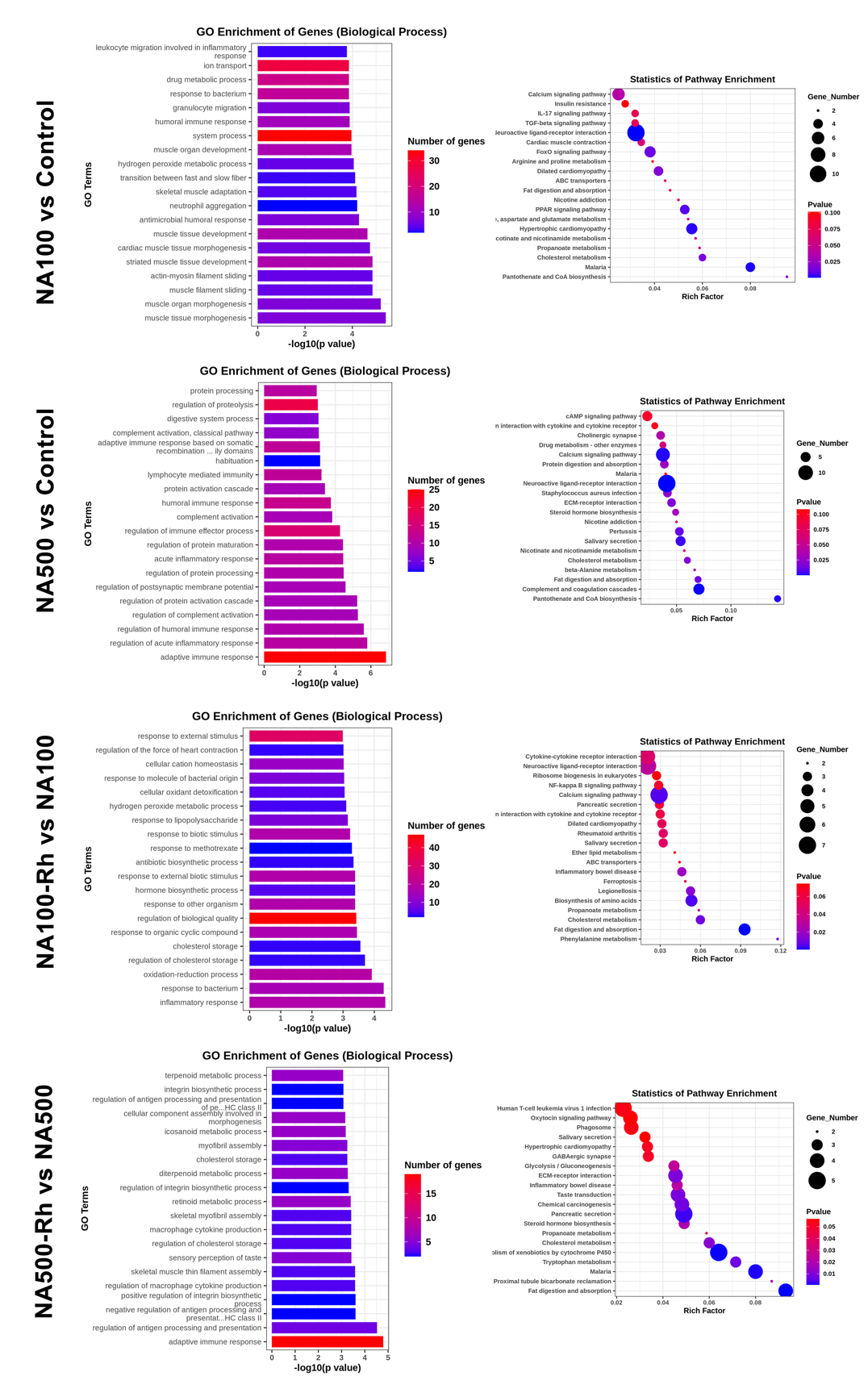
**

**Fig. S3** Go term and KEGG enrichment analysis of the top 20 pathways in different concentrations of NA-only or with rhynchophylline treated groups. NA100: 100 µM NA treated; NA500: 500 µM NA treated; NA100-Rh: 100 µM NA and 100 µM rhynchophylline treated; NA500-Rh: 500 µM NA and 100 µM rhynchophylline treated.
